# Haloperidol Affects Plasticity of Differentiated NG-108 Cells Through σ1R/IP_3_R1 Complex

**DOI:** 10.1007/s10571-017-0524-y

**Published:** 2017-08-07

**Authors:** Jana Kubickova, Lubomira Lencesova, Lucia Csaderova, Tibor Stracina, Sona Hudecova, Petr Babula, Eva Rozborilova, Marie Novakova, Olga Krizanova

**Affiliations:** 1 Institute of Clinical and Translational Research, Biomedical Research Center, Slovak Academy of Sciences, Dubravska cesta 9, 84505 Bratislava, Slovakia; 2 Institute of Virology, Biomedical Research Center, Slovak Academy of Sciences, Bratislava, Slovakia; 3 Department of Physiology, Faculty of Medicine, Masaryk University, Brno, Czech Republic; 4 Clinics of Pneumology and Phthisiology, Jessenius Faculty of Medicine, Martin, Slovakia

**Keywords:** BD 1047, Haloperidol, Inositol 1,4,5-trisphosphate receptor, NG-108 cells, Sigma 1 receptor, Dopamine 2 receptor

## Abstract

Haloperidol is an antipsychotic agent that primarily acts as an antagonist of D2 dopamine receptors. Besides other receptor systems, it targets sigma 1 receptors (σ1Rs) and inositol 1,4,5-trisphosphate receptors (IP_3_Rs). Aim of this work was to investigate possible changes in IP_3_Rs and σ1Rs resulting from haloperidol treatment and to propose physiological consequences in differentiated NG-108 cells, i.e., effect on cellular plasticity. Haloperidol treatment resulted in up-regulation of both type 1 IP_3_Rs (IP_3_R1s) and σ1Rs at mRNA and protein levels. Haloperidol treatment did not alter expression of other types of IP_3_Rs. Calcium release from endoplasmic reticulum (ER) mediated by increased amount of IP_3_R1s elevated cytosolic calcium and generated ER stress. IP_3_R1s were bound to σ1Rs, and translocation of this complex from ER to nucleus occurred in the group of cells treated with haloperidol, which was followed by increased nuclear calcium levels. Haloperidol-induced changes in cytosolic, reticular, and nuclear calcium levels were similar when specific σ1 blocker -BD 1047- was used. Changes in calcium levels in nucleus, ER, and cytoplasm might be responsible for alterations in cellular plasticity, because length of neurites increased and number of neurites decreased in haloperidol-treated differentiated NG-108 cells.

## Introduction

Haloperidol is a typical antipsychotic agent used in the treatment of psychiatric disorders, including various psychoses such as schizophrenia and severe agitated delirium. Several adverse effects of haloperidol treatment are reported among them extrapyramidal side effects such as dystonia and muscle rigidity, palpitations, and changes of arterial blood pressure are common; QT interval prolongation eventually followed by cardiac arrhythmias such as Torsade de Pointe, are also reported (Remijnse et al. 2002).

Haloperidol exhibits high-affinity dopamine D2 receptor (D2R) antagonism. D2Rs play an important role in pathophysiology of brain signaling. These receptors exist as monomeric units, but they can also form oligomers. D2 receptors are associated with Gi proteins to inhibit production of the cAMP. Nevertheless, recently, it was suggested that an imbalance of D1R/D2R heteromers could be related to depressive symptoms in youngsters (Corrales et al. 2016). Putative D1/D2 receptor heterodimers have been suggested to regulate diacylglycerol and IP_3_ signaling by activating Gq (Rashid et al. 2007). It appears that D1 and D2 receptors are both necessary for this pathway; thus, the application of dopamine or a combination of two selective D1 and D2 receptor agonists is able to increase intracellular calcium, whereas treatment with either D1 or D2 receptor antagonist can abolish this effect (Hasbi et al. 2009).

Haloperidol is also known as a ligand of type 1 sigma receptors (σ1Rs). The σ1Rs were first discovered in the central nervous system (Martin et al. 1976) and later, their presence was shown in various tissues (Su and Junien 1994), including heart muscle (Dumont and Lemaire 1991; Novakova et al. 1995). The σ1Rs are non-opioid transmembrane proteins located at the ER, mitochondrial, and plasma membranes (Hayashi and Su 2007). Several in vivo and in vitro studies have shown that overexpression of the σ1R or activation of σ1R by high-affinity ligands protect against neuronal cell death (Martin et al. 2004; Bucolo et al. 2006; Dun et al. 2007; Smith et al. 2008; Tchedre et al. 2008; Zhang et al. 2011). Tchedre and Yorio (2008) reported that in vitro σ1R ligands regulate levels of intracellular Ca^2+^ concomitantly with the attenuated activation of pro-apoptotic genes. Increasing σ1R in vitro counteracts the ER stress response, whereas decreasing σ1R enhances apoptosis (Hayashi and Su 2007). Upon ER-Ca^2+^ depletion or ligand stimulation, σ1Rs dissociate from BiP/GRP78, leading to prolonged Ca^2+^ signaling into the mitochondria via inositol IP_3_Rs. Previously, we have shown that in isolated rat cardiomyocytes, σ1Rs are coupled to type 1 and type 2 IP_3_Rs (Novakova et al. 2007), since silencing of these receptors attenuated expression of the σ1R. Type 3 IP_3_R is also associated with σ1R (Hayashi and Su 2001). It has been proposed that in this complex, σ1R protects IP_3_R from degradation, whereas IP_3_R facilitates the transfer of calcium into the mitochondria and favors cell survival (Kiviluoto et al. 2013).

Because σ1Rs bind to a broad range of synthetic compounds including antipsychotics, they are thought to be potential therapeutic targets for mental disorders; furthermore, σ1Rs might play a pivotal role in neuroprotection (Hayashi and Su 2007; Katnik et al. 2006). Mitsuda and co-workers (2011) have shown that a transcription factor, ATF4, which is considered to be a marker of ER stress, directly binds to the 5′ upstream region of σ1R and modulates its expression. Additionally, the knock-down of ATF4 results in a decrease in the level of σ1R expression. Thus, ER stress, which deeply involves IP_3_Rs, is likely to be a potent modulator of σ1Rs acting through the ATF4 transcription factor.

We hypothesized that haloperidol might affect plasticity of neuronal cells by modulating predominantly σ1Rs and IP_3_Rs, but also D2 receptors. As a model of neuronal cells we used NG-108 stable cell line differentiated by cAMP to the neuronal phenotype (Kubickova et al. 2016). We proposed that mutual interaction/communication of these three receptors in the presence of haloperidol might alter calcium fluxes and change the plasticity of differentiated NG-108 cells.

## Materials and Methods

### Cell Culture

The neuroblastoma-glioma cell line NG-108 (PAA Laboratories, Germany, provided by Dr. Lacinova) was used in these experiments. This line was formed by fusing mouse N18TG2 neuroblastoma cells with rat C6-BU-1 glioma cells in the presence of inactivated Sendai virus (Hamprecht 1977). Cells were plated at relatively low density (0.65 × 10^4^ cells/cm^2^), cultivated for 24 h and differentiated with dibutyryl cAMP (dbcAMP; Sigma, USA) as described in Kubickova et al. (2016). After differentiation, these cells are accepted as a model of neuronal cells.

Unless stated, NG-108 cells were treated with the prototypical σ1R ligand haloperidol (H; 10 µmol/L; Sigma Aldrich, USA) or the specific σ1R antagonist BD 1047 (BD; 10 µmol/L; Sigma Aldrich, USA). Also, some groups of cells were treated with a specific σ1R agonists SA4503 (1 µmol/L; Tocris Bioscience, UK) or PRE-084 (PRE; 1 µmol/L; Tocris Bioscience, UK). To study the mutual interactions between σ1Rs and IP_3_R1, the IP_3_R blocker Xestospongin C (Xest; 1 µmol/L; Calbiochem, USA) was applied. Additionally, combinations of haloperidol or BD 1047 with Xest were used. Transfection of siRNAs was performed as described in Lencesova et al. (2013) using ON-TARGET plus SMART pool σ1R’s siRNA, ON-TARGET plus SMART pool ITPR1’s siRNA, ON-TARGET plus SMART pool ITPR3’s siRNA, and as a control ON-TARGET plus Non-targeting siRNAs (Dharmacon, Thermo Scientific, USA).

### RNA Isolation, cDNA Preparation, PCR, and Real-Time PCR

Total RNA was isolated by the TRI Reagent (MRC Ltd., Cincinnati, OH, USA) as described in Markova et al. (2014). Reverse transcription was performed using 1.5 μg of total RNA and Ready-To-Go You-Prime First-Strand Beads (GE Healthcare Life Sciences, Germany) with the pd(N6) primer. PCR specific for a rat IP_3_R1 (GI 1055286), IP_3_R2 (GI 13752805), and σ1R (GI 38541100) was performed as described in Novakova et al. (2010). Following primers for rat IP_3_R3 (GI 6981109) and β-actin (GI 42475962; as a housekeeping gene control for the semi-quantitative evaluation of PCR) were used: IP_3_R3 forward: 5′-CTGCCCAAGAGGAGGAGGAAG-3′, IP_3_R3 reverse: 5′-GAACAGCGCGGCAATGGA GAAG-3′; RBA1 5′-AGTGTGACGTTGACATCCGT-3′ and RBA2 5′-GACTGATCG TACTCCTGCTT-3′. We also amplified the following genes, which are markers for ER stress: rat ATF4 (GI 165971604), forward 5′-GGCCACCATGGCGTATTAAGA-3′ and reverse 5′- GACATTAAGTCCCCCGCCAA-3′; rat CHOP (GI 203356), forward 5′-AGGGCTAGCTTGGTCCTA GA-3′ and reverse 5′-CCCCAAGTCCTGAACTCCAC-3′; and rat XBP1 spliced form (GI 51948391), forward 5′-TTACGAGAGAAAACTCATGGGC-3′ and reverse 5′-GGGTCCAACTTGTCCAGAATGC-3′. All PCR products were analyzed on 2% agarose gels, and the intensity of individual bands was evaluated by measuring (PCBAS 2.0 software) the optical density per mm^2^ as compared relative to the band corresponding to β-actin. For the relative quantification by real-time PCR, we used identical primers and RNA. Real-time PCR amplifications were carried out as described in Markova et al. (2014) using the SYBR Green Master Mix with ROX reference dye (Life Sciences, EU).

### Western Blot Analysis

Protein concentration of the lysate was determined by using the method of Lowry (1951). Whole procedure is described in detail in Lencesova et al. (2013). An enhanced chemiluminescence detection system (Luminata™ Crescendo Western HRP Substrate, Millipore) was used to detect the bound antibodies, and the optical density of individual bands was quantified using PCBAS 2.0 software.

To detect σ1R protein, we used a rabbit polyclonal antibody against OPRS1 (AB_881796, Abcam, UK), a synthetic peptide derived from the C-terminal region of rat σ1R peptide that recognizes a band of approximately 25 kDa. To detect IP_3_R1 protein, we used a rabbit polyclonal antibody derived from amino acids 1829–1848 of the cytoplasmic C-terminal domain of human IP_3_R1 (AB_260119, Sigma, USA), which recognizes a band of approximately 240 kDa. This sequence is 100% conserved in human, mouse, and rat IP_3_R1.

### Immunoprecipitation

The appropriate monoclonal (3 µg) or polyclonal antibody (6 µg) was incubated with 60 µl of washed magnetic beads (Dynabeads M-280 coated with sheep anti-mouse IgG or M-280 coated with sheep anti-rabbit IgG (Life Technologies, Dynal AS, Norway)) overnight at 4 °C on a rotator (VWR International, LLC, PA, USA). The beads with attached antibodies were washed twice (200 µl) with phosphate-buffered saline (PBS supplemented with 1% bovine serum albumin). Proteins were immunoprecipitated from 1 mg of detergent-extracted total protein via their incubation with antibody-bound beads for 4 h at 4 °C. Bead complexes were washed with PTA (4× with 200 µl; 145 mmol/L NaCl, 10 mmol/L NaH_2_PO_4_, 10 mmol/L sodium azide, and 0.5% Tween 20; pH 7.0). Immunoprecipitated proteins were then extracted with 60 µl of 2× Laemmli loading buffer according to the manufacturer’s instructions (Bio-Rad) and boiled for 5 min. The following antibodies were used for immunoprecipitation: rabbit polyclonal antibody to OPRS1 (σ1R; AB_881796, Abcam, UK) and mouse monoclonal antibody to IP_3_R1 (AB_212025, Calbiochem, Merck Biosciences, Germany).

### Immunofluorescence

Cells grown on glass coverslips were fixed in ice-cold methanol. Nonspecific binding was blocked by incubation with PBS containing 3% bovine serum albumin (BSA) for 60 min at 37 °C. The cells were then incubated with primary antibody diluted 1:500 in PBS with 1% BSA (PBS–BSA) for 1 h at 37 °C. A rabbit polyclonal antibody (AB_212026, Calbiochem, Merck Biosciences, Darmstadt, Germany) directed against 1829–1848 amino acid residues from human IP_3_R1 was used. Another group of cells was incubated with rabbit polyclonal antibody anti-OPRS1 (AB_881796, Abcam, USA) directed against a synthetic peptide derived from the C-terminal region of rat σ1 peptide. Afterwards, the cells were washed three times with PBS/BSA for 10 min, incubated with CF488A goat anti-rabbit IgG (AB_10559670, Biotium) diluted 1:1000 in PBS/BSA for 1 h at 37 °C, and washed as described previously. Finally, the cells were mounted onto slides in mounting medium with Citifluor (Agar Scientific Ltd., Essex, UK) and analyzed by laser scanning confocal microscopy (LSM 510 MetaMicroscope, Zeiss). Images were taken with a Plan Neofluar 40×/1.3 oil objective. Images were scanned at scan speed 7 (260 Hz line frequency), 1024 × 1024 pixels, 12 bit data depth in the average mode (4× line) at optical zoom 3. The Z-stack interval was 0.8 µm. Images of all samples were acquired with the same microscope setup.

### Proximity Ligation Assay (PLA)

PLA was used for the in situ detection of the interaction between D1 and D2 receptors and also between σ1Rs and IP_3_R1s. The assay was performed in a humidified chamber at 37 °C according to the instructions of the manufacturer (Olink Bioscience, Sweden). For this method, following antibodies were used: rabbit polyclonal antibody to OPRS1 (σ1R; AB_881796, Abcam, UK), mouse monoclonal antibody to IP_3_R1 (AB_212025, Calbiochem, Merck Biosciences, Germany), mouse monoclonal antibody to dopamine receptor D1 (SG2-D1α, ab78021, Abcam, UK), and rabbit polyclonal antibody to dopamine receptor D2 (ab21218, Abcam, UK).

### Cytosolic [Ca^2+^]_i_ Staining by Fluo-3AM Fluorescent Dye

For this method we used a fluorescent dye Fluo-3AM (Sigma Aldrich, USA). Method is described in detail in Kubickova et al. (2016).

### Determination of Reticular Calcium by Rhod-5 N

A detailed protocol has been described by (Lencesova et al. 2013). Rhod-5 N fluorescent dye (Invitrogen Ltd., Paisley, UK) was added to each sample to a final concentration 20 μmol/L, and measurements were taken using a BioTek fluorescent reader (excitation 551 nm/emission 576 nm). The results are expressed in arbitrary units.

### Determination of Nuclear Calcium by Rhod-5 N

After 24 h of treatment, cells were gently collected from flasks, allowed to settle, and washed with 1× PBS solution. Gentle lysis was performed with 500 µl of cell lysis buffer from a kit for cytoplasmic and nuclear protein isolation (ProteoJetTM Fermentas, Germany) and 1,4-dithiothreitol to a final concentration of 1 mmol/L. The isolation of cell nuclei was performed according to the kit manufacturer’s instructions. Pellets from the nuclear fraction were homogenized in 200 µl of nuclear lysis buffer from the ProteoJetTM kit and pipetted into a 24-well plate. For each sample, Rhod-5 N fluorescent dye was added to a final concentration of 20 μmol/L, and measurements were taken using a BioTec fluorescent reader (BioTec, Germany) at 551 nm (excitation) and 576 nm (emission). After the fluorescence was measured, the signal was quenched by adding EGTA solution (pH 7.0) to final concentrations of 0.25, 1.0, 2.5, and 5.0 mmol/L. The results are expressed in arbitrary units.

### Quantification of Neurite’s Outgrowth

Neurite outgrowth was determined as described in Kubickova et al. (2016). Quantification of neurite outgrowth was verified by “Neurite Outgrowth Staining Kit”. To visualize cell viability and neurite’s outgrowth we used a dual-color stain (Life Technologies, Dynal AS, Norway). For our experiments, we used combination of the cell viability indicator and the cell membrane stain (diluted 1000-fold) in Dulbecco’s Phosphate-Buffered Saline (DPBS, Thermo Fisher Scientific, Hampshire, UK) containing calcium and magnesium. Neurite outgrowth was analyzed by laser scanning confocal microscopy (LSM 510 MetaMicroscope, Zeiss) and also by BioTek fluorescence scanner (BioTek, Germany), where quantification of a relative fluorescence was performed. Indicator of cell viability was measured using excitation/emission wavelengths of 483/525 nm and cell membrane stain was measured at excitation/emission wavelengths of 554/567 nm. The results were expressed as arbitrary units.

### Statistical Analysis

Each value represents an average of 3–9 wells from at least two independent cultivations of NG-108 cells. The results are presented as the mean ± S.E.M. Significant differences between the groups were determined by one-way ANOVA. For multiple comparisons, an adjusted *t* test with *p* values corrected by the Bonferroni method was used.

## Results

**Fig. 1:**
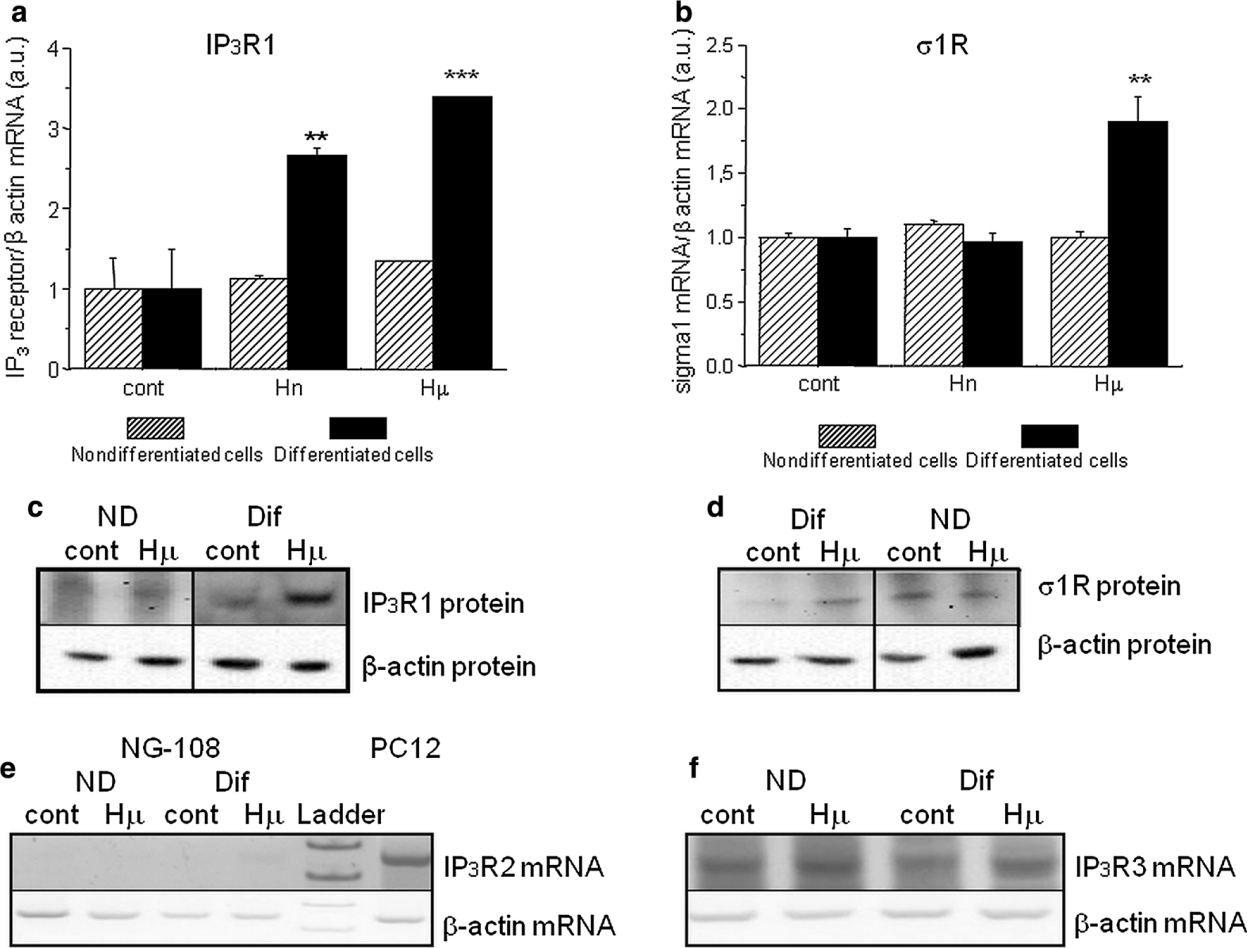
Haloperidol increases the mRNA (**a**, **b**) and protein (**c**, **d**) levels of σ1R (**b**, **d**) and type 1 (**a**, **c**), but not type 2 (**e**) and 3 (**f**), IP_3_ receptors in differentiated NG-108 cells (*Dif*). In contrast to non-differentiated (*ND*) cells (**a**, **b** *striped columns*), in differentiated cells (**a**, **b** *black columns*) haloperidol at a concentration of 10 nmol/L (*low-dose*; *Hn*) increases the mRNA expression of IP_3_R1, and at a concentration of 10 µmol/L (*high-dose*; *Hμ*), the mRNA and protein expression of both IP_3_R1 and σR1 was increased (**a**, **b**). Type 2 IP_3_ receptors are not expressed in NG-108 cells (**e**); we observed an expression signal in PC12 cells but not in NG-108 cells. The mRNA expression of type 3 IP_3_ receptors was not changed in undifferentiated or in differentiated cells following Hμ treatment (**f**). The results are expressed as the mean ± SEM. Statistical significance: **p < 0.01 and ***p < 0.0001 compared to control untreated cells

**Fig. 2:**
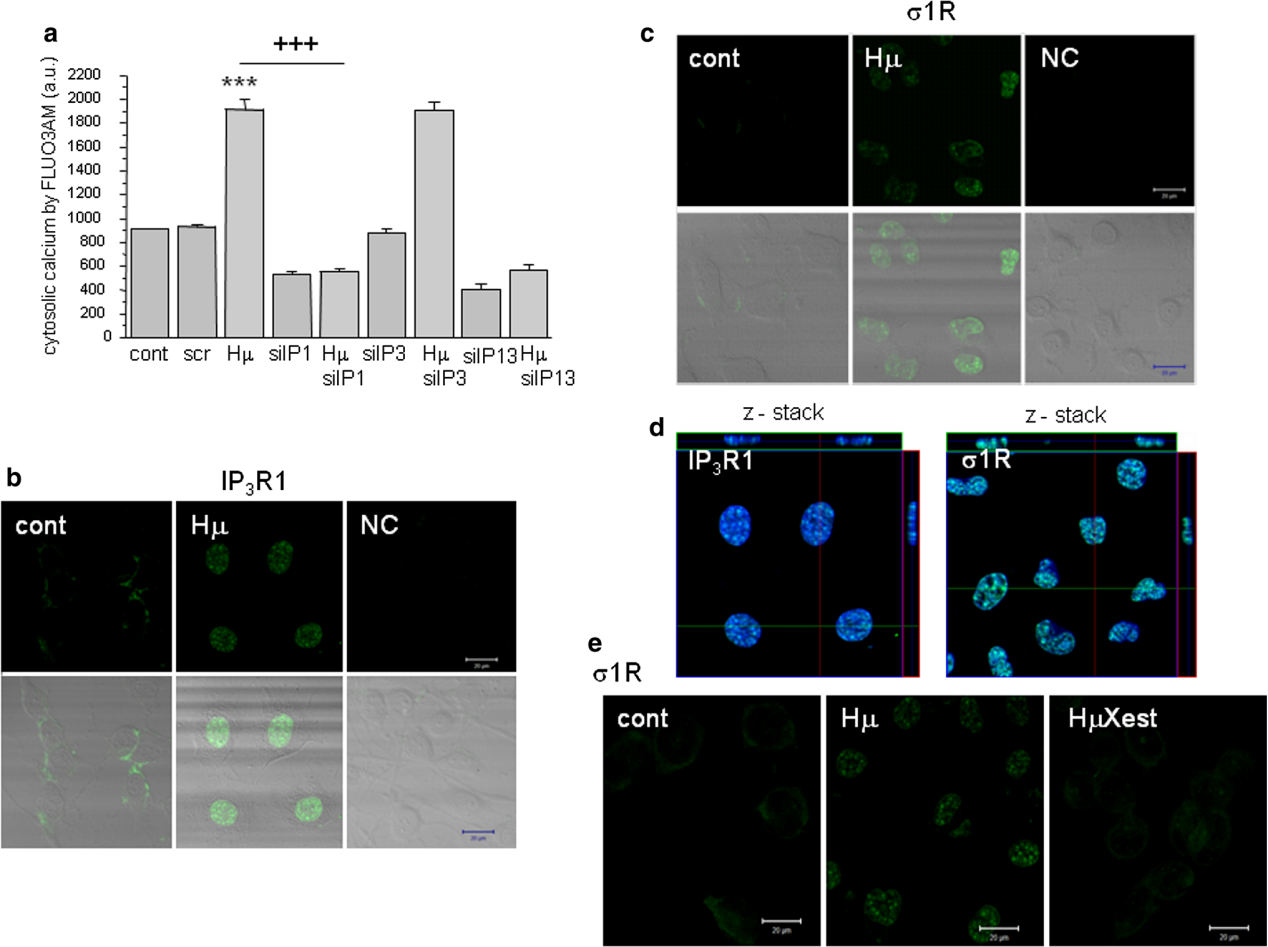
Involvement of IP_3_R1, but not IP_3_R3 in haloperidol-induced changes in levels of cytosolic calcium and translocation of the IP_3_R1 and σ1R to the nucleus following haloperidol treatment. Experiments were performed on NG-108 cells differentiated by dbcAMP. To determined haloperidol-induced changes in cytosolic calcium due to IP_3_R1/IP_3_R3 receptors, these receptors were silenced either individually, or both of them and levels of cytosolic calcium levels were determined after haloperidol treatment (**a**). Silencing of IP_3_R1, but not IP_3_R3 caused significant decrease in cytosolic calcium levels, thus proving involvement of the IP_3_R1, but not IP_3_R3 in haloperidol-induced increase in cytosolic calcium. In control cells (*cont*), IP_3_R1s (**b**, *green signal*) and σ1Rs (**c**, *green signal*) are localized to the ER. Following haloperidol treatment (*Hμ*; 10 µmol/L), these receptors translocate to the nucleus (**b**, **c**, *green signal*). Translocation of the IP_3_R1 and σ1R was verified by z-stacks from the Hμ-treated cells (**d**), which clearly shows an intranuclear signal. In the presence of Xestospongin C (*Xest*; 1 µmol/L), the Hμ -induced translocation of σ1R does not occur (**e**, *HμXest*). Results in the graph are expressed as the mean ± SEM and represent an average of six parallels from two independent cultivations. Statistical significance compared to control was ***p < 0.0001 and compared to Hμ treated cells was +++*p* < 0.0001

**Fig. 3:**
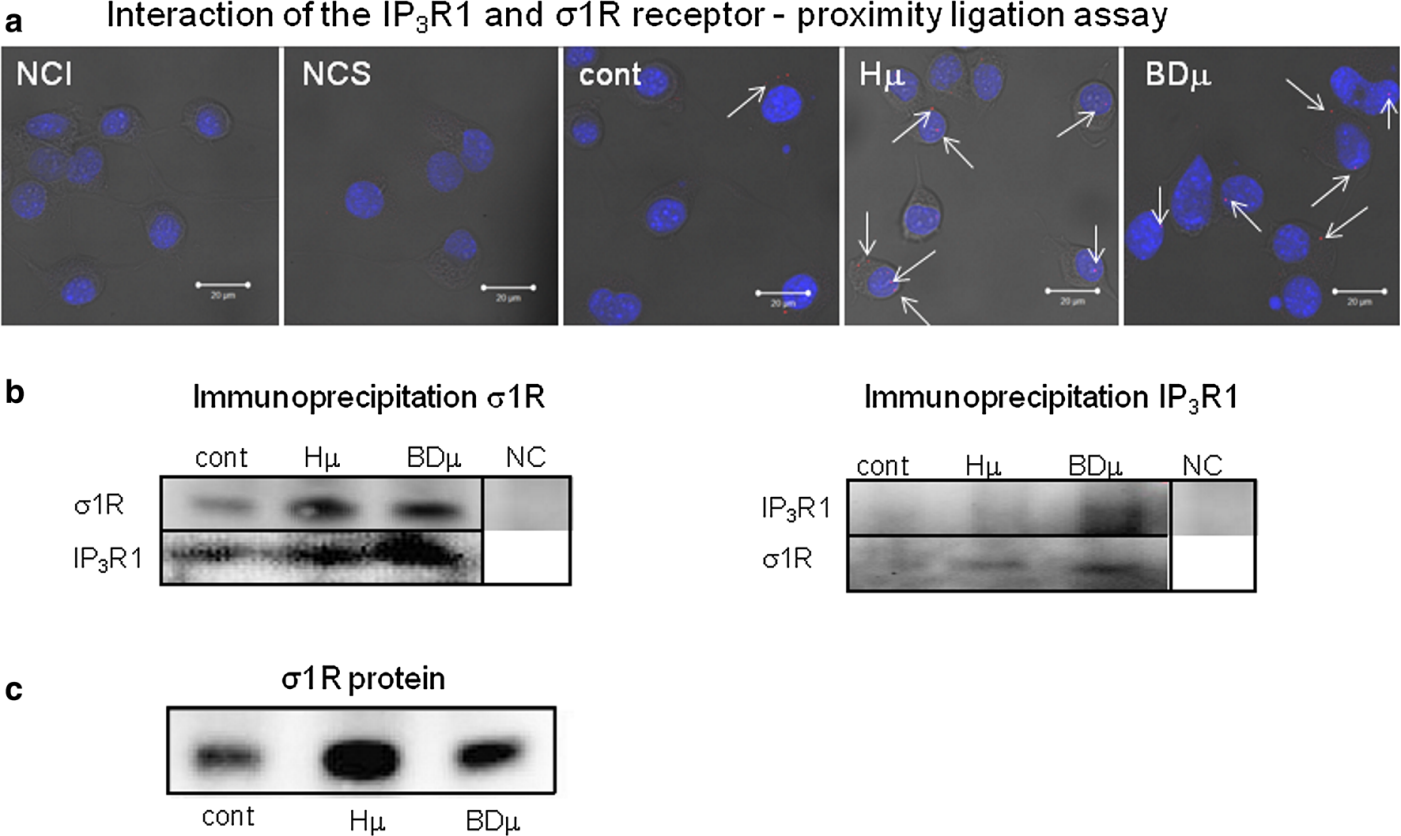
Interaction of the IP_3_R1 and σ1R receptor was verified by proximity ligation assay and immunoprecipitation of these receptors. Experiments were performed on NG-108 cells differentiated by dbcAMP. The mutual interaction of the IP_3_R1 and σ1R was verified by a proximity ligation assay (**a**), where *red dots* show co-localization of these two receptors. *Bar* represents 20 µm. *NCI* negative control without IP_3_R1 primary antibody, *NCS* negative control without σ1R primary antibody. Immunoprecipitated σ1Rs bound the IP_3_R1 in control cells (**b**; *cont*) and in cells treated with Hμ or BDµ (**b**). In agreement, immunoprecipitated IP_3_R1 bound σ1Rs in Hμ- and BDµ-treated cells (**b**). Western blot analysis documented amount of the σ1R protein in control cells, Hμ treated cells and BD 1047 (*BDµ*; 10 µmol/L) treated cells (**c**)

In differentiated NG-108 cells, we observed a concentration-dependent increase in IP_3_R1 mRNA (Fig. 1a; black columns) and in σ1R (Fig. 1b; black columns), while in non-differentiated cells, no changes in the corresponding mRNA (Fig. 1a, b; striped columns) or protein (Fig. 1c, d) were visible. In differentiated cells, treatment with haloperidol at a concentration of 10 nmol/L (Hn) for 24 h increased IP_3_R1 mRNA levels from 1.0 ± 0.4 a.u. to 2.7 ± 0.1 a.u. (**p < 0.01), while the mRNA levels of σ1R were increased by haloperidol treatment (from 1.0 ± 0.1 a.u. to 1.9 ± 0.2 a.u., **p < 0.01) only at the concentration of 10 µmol/L (Hμ). Additionally, we observed a significant increase in protein expression of IP_3_R1 (Fig. 1c) and σ1R (Fig. 1d) after 24 h of Hμ treatment. The expression of the type 3 IP_3_R was unchanged following this treatment (Fig. 1f). IP_3_R2s are not expressed in differentiated NG-108 cells (Fig. 1e), as verified using the PC12 cell type, where a clear signal of the IP_3_R2 was visible. We proposed that increased level of IP_3_R1 due to Hμ treatment might be responsible for increased levels of cytosolic calcium. Therefore, we silenced IP_3_R1, IP_3_R3, or combination of both and determined levels of cytosolic calcium with/without Hμ treatment (Fig. 2a). Silencing of the IP_3_R1 or IP_3_R1/IP_3_R3 followed by Hμ treatment resulted in decreased levels of cytosolic calcium, thus proving involvement of this receptor in Hμ-induced increase of cytosolic calcium (Fig. 2a). Silencing of the IP_3_R3 and Hμ treatment did not change calcium levels compared to Hμ treated group. In control cells, the IP_3_R1 was localized to the endoplasmic reticulum, but after Hμ treatment; we observed the translocation of IP_3_R1 from the ER to the nucleus (Fig. 2b; green sinal) and translocation of σ1Rs to the nucleus as well (Fig. 2c; green signal). To further verify the translocation of IP_3_R1 and σ1Rs, we obtained confocal z-stacks from the images that confirmed a positive signal in the nucleus (Fig. 2d). Following simultaneous incubation with Hμ and Xest (1 µmol/L), σ1Rs remain localized primarily to the ER (Fig. 2e). Co-localization of IP_3_R1 with σ1Rs was determined by proximity ligation assay (Fig. 3a) and immunoprecipitation (Fig. 3b). By immunoprecipitation, we clearly showed that IP_3_R1 co-immunoprecipitates with σ1Rs (Fig. 3b; left) in control cells and in Hμ and BDµ-treated cells. Reverse immunoprecipitation with IP_3_R1 resulted in the co-immunoprecipitation of σ1Rs (Fig. 3b; right), further demonstrating the clustering of these receptors. Negative controls verified the specificity of the immunoprecipitation. This observation was verified by a proximity ligation assay, where red dots showing the interaction of these two receptors were observed (Fig. 3a). Since haloperidol is a nonspecific ligand of σ1Rs, we compared the results observed following haloperidol treatment with those from a specific blocker of σ1Rs, BD 1047 at a concentration of 10 µmol/L (BDµ). Western blot analysis documented the higher amount of σ1R protein in Hμ treated cells and BDµ treated cells compared to untreated control cells (Fig. 2e).

**Fig. 4:**
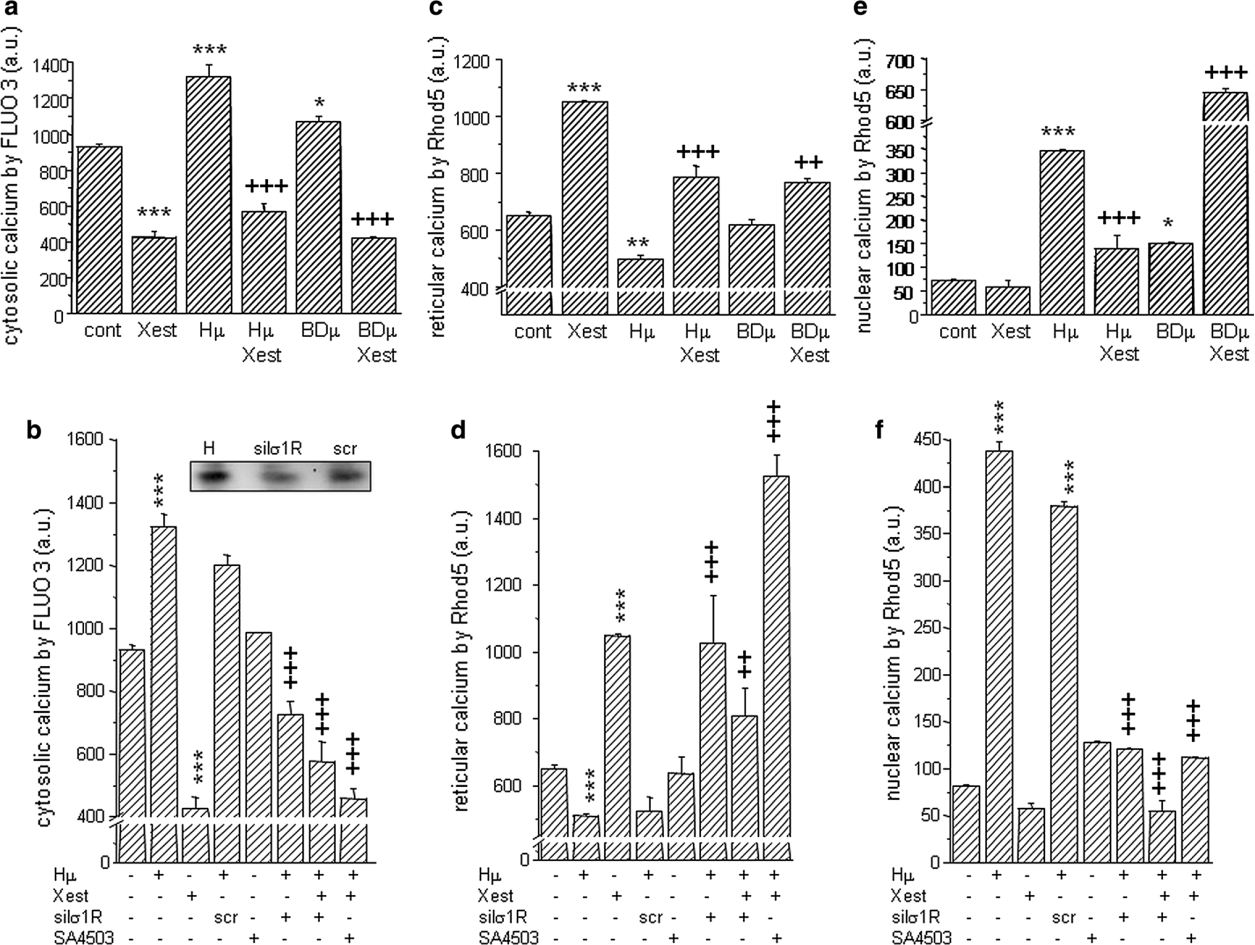
Relative levels of cytosolic (**a**, **b**), reticular (**c**, **d**) and nuclear (**e**, **f**) calcium in differentiated NG-108 cells treated with haloperidol (*Hμ;* 10 µmol/L), BD 1047 (*BDµ*; 10 µmol/L), the IP_3_R blocker Xestospongin C (*Xest*; 1 µmol/L), and the combination of Xest with Hμ or BDµ (*comb*) for 24 h (**a**, **c**, **e**). Combination of Hμ with SA4503 (1 µmol/L) and also with silenced σ1R was used (**b**, **d**, **f**). As a control serves scrambled siRNA (*scr*). Hμ treatment significantly increased the levels of cytosolic calcium, while Xest treatment in combination with Hμ completely prevented this increase (**a**). Silencing of the σ1R in Hμ-treated cells significantly decreased a level of cytosolic calcium compared to *scr* or plain Hμ-treated cells (**b**). The σ1R-agonist SA4503 further decreased a level of cytosolic calcium compared to control cells (**b**). Accordingly, reticular calcium was decreased in Hμ-treated cells compared to control cells, but Xest, SA4503 treatment, or silencing of the σ1R increased a level of reticular calcium compared to Hμ-treated cells (**c**, **d**). The results observed following BDµ treatment were similar to those following Hμ treatment. In nuclei, Hμ increased nuclear calcium level, which was decreased by Xest, SA4503, or silencing of the σ1Rs (**e**, **f**). Surprisingly, when cells were treated in parallel with BDµ and Xest, huge increase in nuclear calcium level was observed compared to plain BDµ-treated cells (**e**). Each column represents an average of six independent cultivations and is displayed as the mean ± S.E.M. Statistical significance compared to controls is * p < 0.05, ** p < 0.01, *** p < 0.001, and compared to the haloperidol group is ++*p* < 0.01 and +++*p* < 0.001

A significant increase in cytosolic calcium was observed in Hμ-treated cells compared to untreated controls (Fig. 4a; from 933 ± 14 a.u. to 1323 ± 64 a.u., ***p < 0.0001). A rapid decrease in cytosolic calcium occurs when cells were treated with both Hμ and the IP_3_R blocker Xest (567 ± 48 a.u., +++*p* < 0.0001). Also, we observed a significant increase in cytosolic calcium following treatment with BDµ and a rapid decrease when cells were treated in parallel with BDµ and Xest (Fig. 4a; from 1070 ± 27 a.u. to 421 ± 10 a.u., +++*p* < 0.0001). Involvement of σ1Rs in the Hμ-induced increase of the cytosolic calcium was verified by σ1Rs silencing using appropriate siRNA (Fig. 4b). Hμ-induced increase of the cytosolic calcium level was prevented also by a parallel treatment with Xest or a specific σ1Rs agonist SA4503 (1 µmol/L) (Fig. 4b). Modulation of cytosolic calcium by the IP_3_R blocker Xest suggests a release of reticular calcium stores. Thus, we measured reticular calcium in Hμ and BDµ-treated cells (Fig. 4c) and we observed that in both cases, the level of reticular calcium decreased following a 24-h treatment (from 651.0 ± 12.8 a.u. to 500.2 ± 10.8 a.u. (Hμ), **p < 0.01; or 617.4 ± 21.3 a.u. (BDµ), and was significantly increased when Xest was added in parallel (785.1 ± 40.4 a.u. (Hμ); or 765.3 ± 15.0 a.u. (BDµ)). Silencing of the σ1Rs mRNA in Hμ-treated cells resulted in an increase of the reticular calcium, similarly as SA4503 (Fig. 4d). Interestingly, huge increase in reticular calcium compared to untreated cells occurs, when Hμ-treated cells were incubated in parallel with both, Xest and SA4503 (Fig. 4d). Because Hμ treatment results in the translocation of both IP_3_R1 and σ1Rs to the nucleus, we measured nuclear calcium levels in isolated nuclei (Fig. 4e). Both Hμ and BDµ treatments significantly increased the level of nuclear calcium after 24 h (from 73.5 ± 1.1 a.u. to 345.5 ± 3.9 a.u. (Hμ), ***p < 0.0001; or 151.0 ± 2.1 a.u. (BDµ), *p < 0.05). Xest decreased the level of nuclear calcium in Hμ-treated cells but surprisingly led to a rapid increase in nuclear calcium levels in BDµ-treated cells (Fig. 4e; 139.6 ± 29.0 a.u. (Hμ); or 645.8 ± 6.6 a.u. (BDµ), +++*p* < 0.0001). In Hμ-treated cells, silencing of the σ1Rs mRNA significantly decreased a level of nuclear calcium compared to plain Hμ-treated cells (Fig. 4f).

**Fig. 5:**
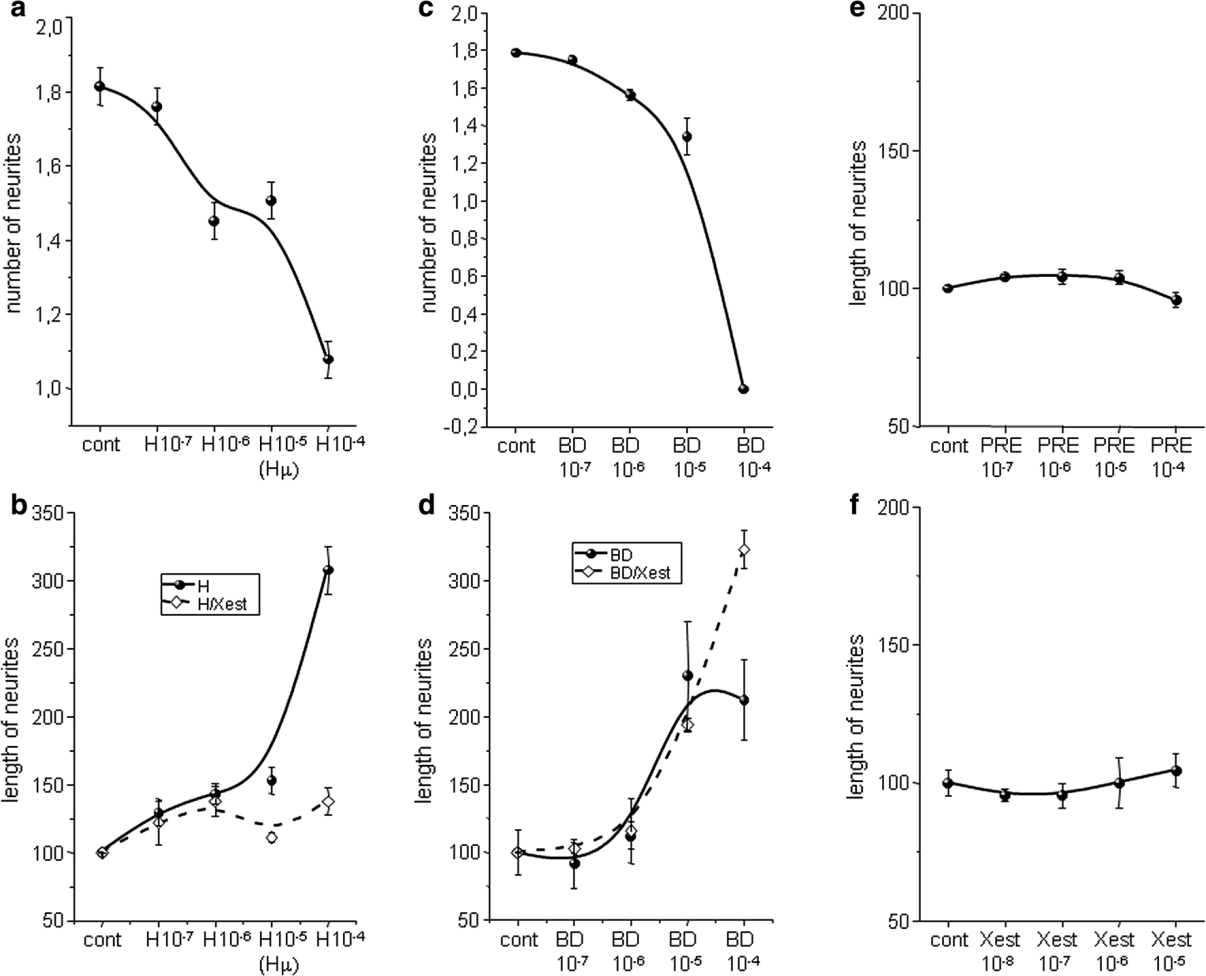
Concentration-dependence of haloperidol, BD 1047, Xestospongin C (*Xest*) and PRE-084 treatment on the number of neurites per cell (**a**, **c**), and neurite outgrowth (**b**, **d**–**f**). Experiments were performed on NG-108 cells differentiated by dbcAMP. Number of neurites in haloperidol-treated cells for 24 h decreased gradually from 0 to 100 µmol/L (**a**), while length of neurites increased (**b**). Length of neurites was lower in cells treated in parallel with haloperidol and Xest (1 µmol/L) (**b**; *dashed*). Similar results were observed with specific σ1R antagonist BD 1047 (**c**, **d**), which was used in the same concentrations as haloperidol. However, the effect of Xest (1 µmol/L) on BD 1047-treated cells varied according to the BD 1047 concentration (**d**). The σ1R agonist PRE-084 (**e**) and IP_3_R blocker Xest (**f**) did not change the length of neurites in a concentration-dependent manner

The physiological impact of these treatments was determined by measuring the number of neurites per cell and neurite outgrowth in cells treated with haloperidol and BD 1047 along with those treatments in combination with the Xest. Haloperidol (H10^−7^–H10^−4^) treatment decreased the number of neurites in a concentration-dependent manner (Fig. 5a). However, length of neurites increased due to a haloperidol treatment in a concentration-dependent manner. This increase was prevented, when haloperidol-treated cells were incubated with Xest in parallel (HXest; Fig. 5b). Similar results were obtained when BD 1047 (BD10^−7^–BD10^−4^) was used (Fig. 5c, d). However, effect of the BD 1047/Xest treatment (BDXest) on the neurite’s outgrowth was highly dependent on a BD 1047 concentration (Fig. 5d). Neither σ1Rs agonist PRE-084 (PRE10^−7^–PRE10^−4^), nor IP_3_R blocker Xest (Xest10^−8^–Xest10^−5^) modulated length of neurites by a concentration-dependent manner (Fig. 5e, f).

**Fig. 6:**
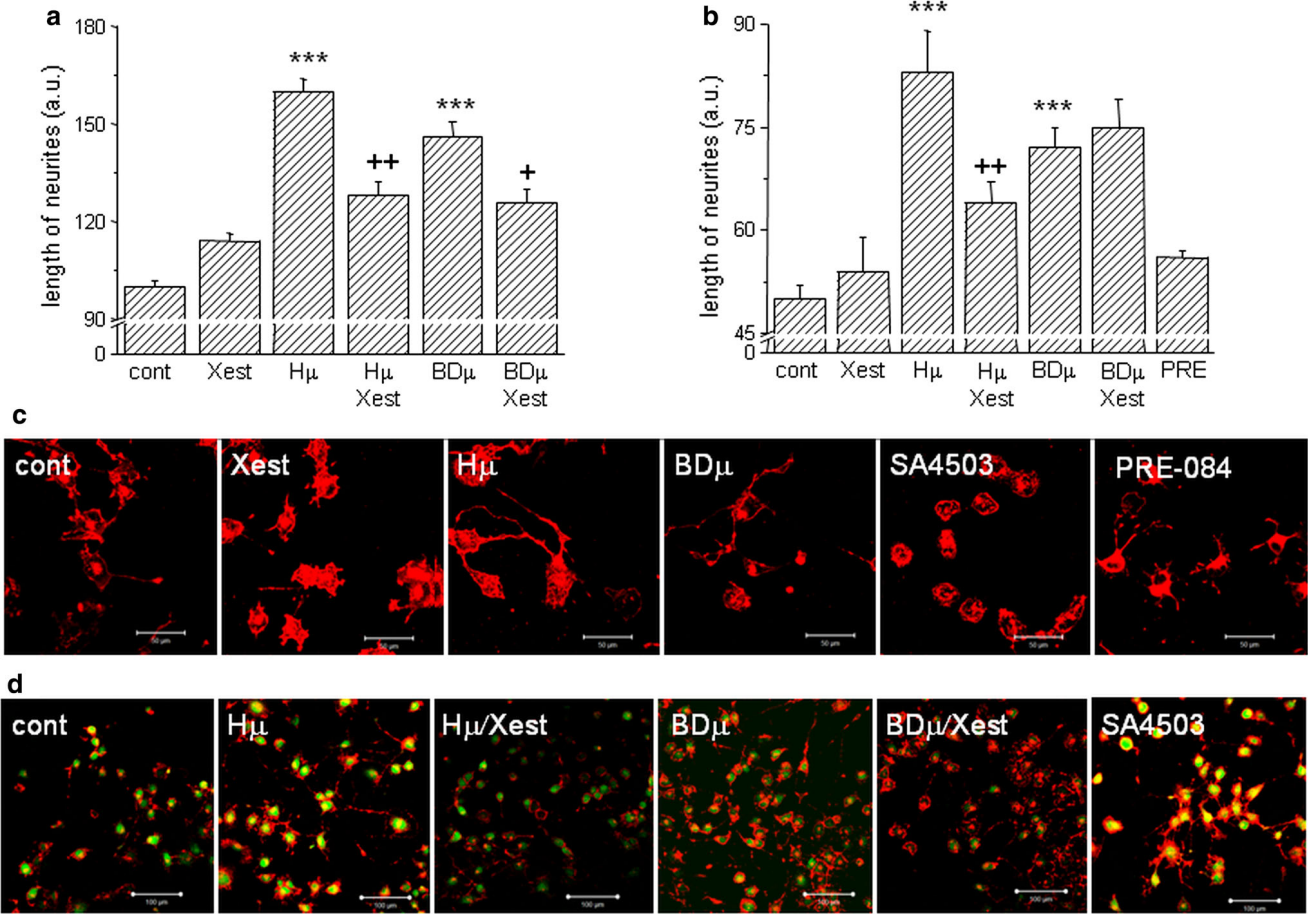
Effect of haloperidol (Hμ) and BD1047 (BDμ) on the length of neurites. Experiments were performed on NG-108 cells differentiated by dbcAMP. The cells were treated for 24 h with Hμ (10 µmol/L), BDμ (10 µmol/L) and Xest (1 µmol/L) after 72 h of differentiation. Length of neurites was measured either manually by ImageJ program (**a**), or using “Neurite outgrowth staining kit” (**b**–**d**). By both methods it is clearly shown that Hμ increased the length of neurites and this increase is partially prevented by Xest. Similar increase in length of neurites was visible after the BDµ treatment, although the effect of Xest was not so conclusive (**a**, **b**). Results from the confocal microscopy without a cell viability staining (**c**; *bar* represents 50 µm) or together with the cell viability stain (**d**; *bar* represents 100 µm) supported results from the fluorescent reader. Each *column* represents an average of 450–835 cells, and the results are displayed as the mean ± S.E.M. Statistical significance compared to controls is *p < 0.05, **p < 0.01, and ***p < 0.001 vs. control and +*p* < 0.05; ++*p* < 0.01, and +++*p* < 0.001 vs. Hμ or BDμ-treated cells

**Fig. 7:**
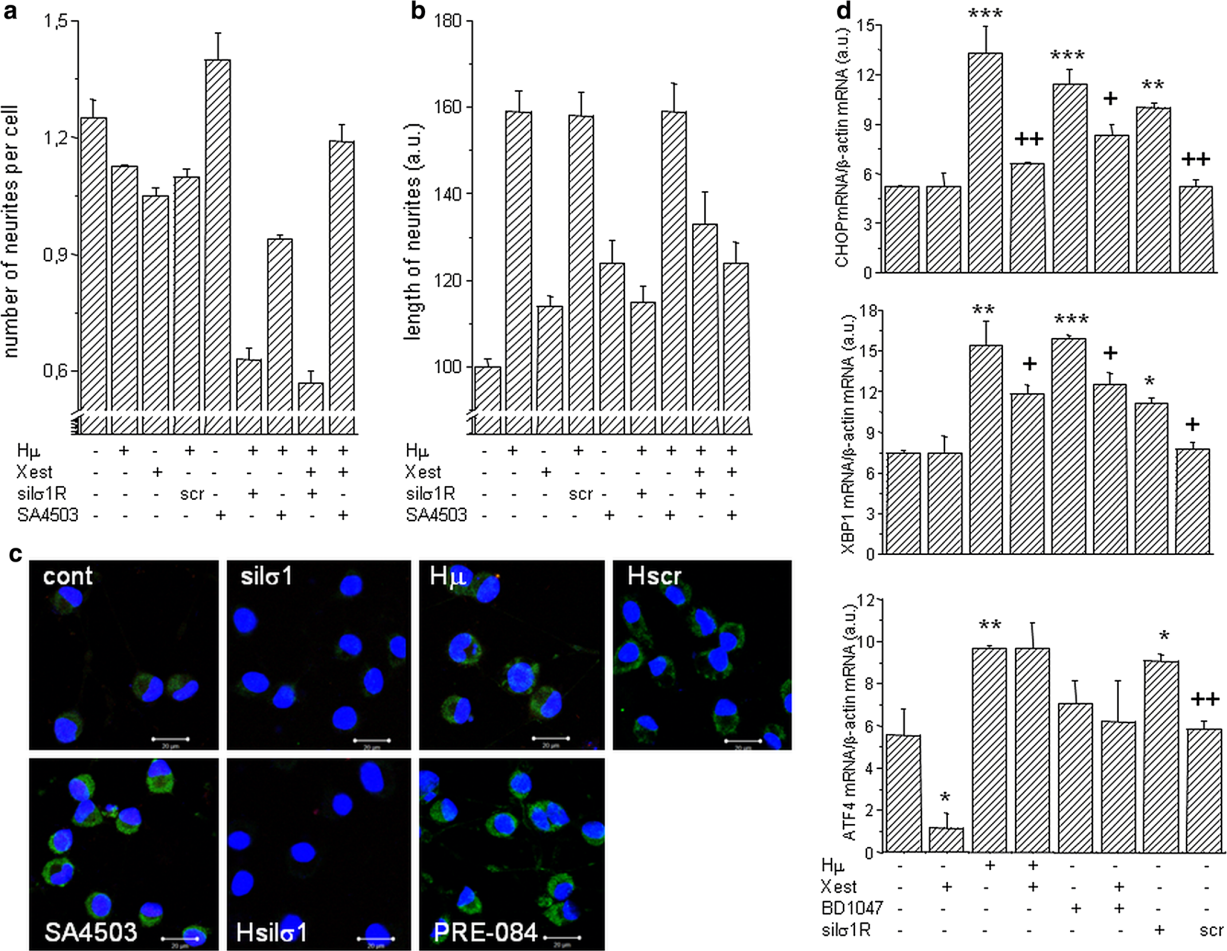
The impact of haloperidol treatment on cell’s plasticity. Experiments were performed on NG-108 cells differentiated by dbcAMP. The pharmacological impact was determined by measuring the number of neurites per cell (**a**) and neurite outgrowth (**b**) in haloperidol (*Hμ*; 10 µmol/L)-treated cells with a parallel treatment of Xestospongin C (*Xest*; 1 µmol/L), SA4503 (1 µmol/L) and with silenced σ1R. Number of neurites decreased significantly in Hμ-treated cells with silenced σ1R (**a**), but not with a scrambled siRNA. Silencing of the σ1R in Hμ-treated cells decreased significantly compared to plain Hμ-treated cell, similarly as by σ1R agonist SA4503 (**b**). Effectivity of σ1R silencing was verified by immunofluorescent staining (**c**). *Bar* represents 20 µm. Induction of markers of ER stress in Hμ, BDμ, and siRNA σ1R -treated differentiated NG-108 cells (**d**). The relative mRNA levels of CHOP, XBP1, and ATF4 were determined in control (*cont*), Hμ-treated and BDμ cells with or without Xest, then in cells after silencing of the σ1R (silσ1R) and scrambled siRNA (*scr*). A significant increase compared to control was observed in silσ1R cells and in Hμ- and BDμ-treated cells, but not in combination of these compounds with Xest. The results are expressed as the mean ± SEM. Statistical significance: * p < 0.05, ** p < 0.01, and *** p < 0.001 compared to untreated control cells; *+ p* < 0.05, *++ p* < 0.01 compared to Hμ, BD, and/or silσ1R treated group

Neurite outgrowth was measured in NG-108 cells differentiated for 72 h and further treated with Hμ, BDµ, and/or Xest, but also with the σ1Rs agonists SA4503 and PRE-084 (10 µmol/L) (Fig. 6). For these measurements, dual approach was used—measuring of individual neurites (Fig. 6a) and evaluation of the fluorescence signal (Fig. 6b, c, d). We observed a significant increase in the length of neurites in Hμ- and BDµ-treated differentiated cells (Fig. 6a, b) compared to untreated control cells. Parallel treatment with Xest decreased partially the length of neurites (Fig. 6a, b). Elevated neurite outgrowth was clearly visible in Hμ and BDµ treated cells compared to control cells, or SA4503 and/or PRE-084 treated cells (Fig. 6c, d; red signal). Green signal shows the viability of cells (Fig. 6d). In order to show the participation of the σ1Rs in the cell plasticity, we silenced these receptors and subsequently measure the number and length of neurites (Fig. 7). Silencing of the σ1Rs in the Hμ-treated cells significantly downregulates number and also length of neurites compared to Hμ-treated cells with or without scrambled siRNA (Fig. 7a, b). Effectivity of the σ1Rs silencing is visible on cell images (Fig. 7c; green signal).

Hμ treatment increased markers of ER stress, CHOP (contr. 5.2 ± 0.1 a.u.; Hμ 13.3 ± 1.6 a.u., ***p < 0.0001), XBP1 (contr. 7.5 ± 0.2 a.u.; Hμ 15.4 ± 1.8 a.u., **p < 0.01), and ATF4 (contr. 5.6 ± 1.2 a.u.; Hμ 9.7 ± 0.1 a.u., **p < 0.01), in differentiated NG-108 cells (Fig. 7d). This increase was also observed when a specific blocker of σ1R, BDµ, was used. Moreover, silencing of the σ1Rs results in an increase of the gene expression of ER stress markers—CHOP, XBP1, and ATF4 (Fig. 7d). Parallel treatment with Xest partially prevented Hμ induced gene expression of CHOP (Hμ 13.3 ± 1.6 a.u.; Hμ/Xest 6.6 ± 0.1 a.u.) or XBP1 (Hμ 15.4 ± 1.8 a.u.; Hμ/Xest 11.8 ± 0.6 a.u.). Unexpectedly, parallel treatment with Hμ and Xest revealed the same ATF4 mRNA levels (Hμ 9.7 ± 0.1 a.u.; Hμ/Xest 9.7 ± 1.2) a.u. as in Hμ treated cells (Fig. 7d).

**Fig. 8:**
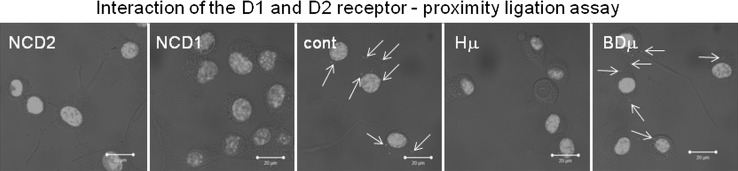
The effect of haloperidol treatment on disintegration of D1/D2 heterodimeric complex in differentiated NG-108 cells. Mutual interaction of the D1 and D2 receptor was verified by a proximity ligation assay, where *red dots* (marked also by *arrows*) show co-localization of these two receptors in control (*Cont*) and BD1047 treated cells (*BDμ*), but not in haloperidol-treated (*Hμ*) group. Bar represents 20 µm. *NCD2* negative control without D2 receptor’s primary antibody, *NCD1* negative control without D1 receptor’s primary antibody

It is known that signaling of D2 receptors is realized through Gi and inhibition of adenylate cyclase, while D1/D2 heterodimeric complex acts through Gq and phospholipase C, which results in the IP_3_ production. Using proximity ligation assay we observed clear co-localization of D1/D2 receptors in differentiated NG-108 control cells (Fig. 8; red dots), but not in haloperidol-treated cells. These results suggest haloperidol-induced disintegration of D1/D2 receptor complex and thus switch from the IP_3_ to cAMP signaling.

## Discussion

In this work, we have clearly shown that in differentiated NG-108 cells haloperidol modulates plasticity of these cells, i.e., decreases number of neurites and increases the length of neurites. Haloperidol-induced changes in cell’s plasticity are probably due to changes in cytosolic and reticular calcium that is modulated by up-regulation of the expression of IP_3_R1. Haloperidol increases expression of both IP_3_R1 and σ1R in differentiated NG-108 cells. Since haloperidol also increases the expression of IP_3_R2s in cardiac atria (Novakova et al. 2010; Tagashira et al. 2013), we investigated the gene expression of type 2 and 3 IP_3_Rs in differentiated NG-108 cells. We observed that these cells do not express IP_3_R2s and that the expression of IP_3_R3s was unaffected by Hμ treatment. Therefore, we focused our interest on the IP_3_R1.

Haloperidol increased cytosolic calcium compared to untreated controls. This increase was abolished by IP_3_R blocker Xestospongin C, which was used in parallel with haloperidol. In agreement, reticular calcium was decreased in cells treated with haloperidol and this decrease was prevented by Xestospongin C. Based on these results, we concluded that haloperidol-induced increased cytosolic calcium is due to calcium depletion from the reticulum. Since haloperidol increased expression of the IP_3_R1 (and not IP_3_R3), we propose that depletion of the reticulum is through the IP_3_R1.

Another question is how σ1Rs contribute to this process. The σ1R antagonist BD1047 increased levels of cytosolic calcium, but did not change reticular calcium levels. However, levels of the nuclear calcium were increased by the treatment with BD1047, although not to the same extent as with haloperidol treatment. These results (together with results from immunofluorescence and proximity ligation assay) would suggest that sigma-1 receptor blocking plays the role primarily in increasing levels of the IP_3_R1s (but not their activity, since reticular calcium was not changed by BD 1047 treatment) and their translocation to the nucleus. This is supported by experiments with silenced σ1R and haloperidol treatment in parallel, where we have observed that in these cells, haloperidol-induced increase in the nuclear calcium was lower in cells, where σ1R was silenced. Also, in the cells where σ1R was silenced, reticular calcium overload was detectable (not shown).

Taken together, we hypothesized that while haloperidol affects expression and activity of the IP_3_R1s, σ1Rs might be more responsible for their trafficking into the nucleus (which in turn might affect expression of the IP_3_R1).

We have shown that Hμ treatment for 24 h causes increase in the expression, complex formation, and translocation of both IP_3_R1s and σ1Rs to the nucleus. The IP_3_R1/σ1Rs complex has already been reported in hepatocytes (Abou-Lovergne et al. 2011). Additionally, it has been shown that σ1Rs affect Ca^2+^ signaling in NG-108 (Hayashi and Su 2001) and MCF-7 cells via the formation of a trimeric complex with ankyrin B and IP_3_R3. In our hands, IP_3_R3s were not affected by Hμ treatment in NG-108 cells; therefore, we focused on the IP_3_R1/σ1Rs complex.

Previously, we have shown that IP_3_R1s aggregate and form intranuclear clusters when cells are treated with certain pro-apoptotic agents (Lencesova et al. 2013; Ondrias et al. 2011). Miki and co-workers (2015) have found intranuclear aggregates of σ1Rs with huntingtin, and they reported that σ1R is involved in the degradation of intranuclear inclusions in a cellular model of Huntington’s disease. Additionally, translocated σ1Rs have been shown to co-localize partially with PML bodies, which are suggested to play a role in transcriptional regulation and nuclear protein sequestration (Spector 2006) and also apoptosis. We propose that translocation of the σ1Rs and IP_3_R1s might alter transcription of certain genes through changes in intranuclear calcium. It has been shown that σ1Rs are involved in the regulation of intracellular [Ca^2+^]_i_ by affecting Ca^2+^-influx or the release from intracellular stores (Gasparre et al. 2012). The Ca^2+^-response triggered by an extracellular ligand engaging the IP_3_/Ca^2+^ pathway can be increased by σ1R agonists and decreased by σ1R antagonists (Gasparre et al. 2012). The σ1Rs could affect Ca^2+^ signaling because it has been shown that σ1R ligands affect Ca^2+^-influx and the beating rate of cardiac myocytes (Ela et al. 1994). We observed that Hμ increased cytosolic calcium levels compared to control untreated cells. At the same time, a decrease in reticular calcium occurs suggesting the depletion of the ER via IP_3_R1. Calcium depletion in the ER is accompanied by ER stress. Indeed, we observed increased markers of ER stress such as ATF4, XBP1, and CHOP in haloperidol-treated group. Involvement of the σ1Rs in ER stress was proved by their silencing and subsequent increased levels of above-mentioned markers. In cancer cells, σ1R antagonists evoke ER stress response that is inhibited by σ1R agonists (Do et al. 2013; Mori et al. 2013; Wang et al. 2012). Omi and co-workers (2014) demonstrated that ER stress induces σ1R expression through the PERK pathway, which is one of the cell’s responses to ER stress. In addition, it has been demonstrated that induction of σ1R can repress cell death signaling. Thus, we propose that ER stress might be a trigger for σ1R overexpression, binding to the IP_3_R1s and translocation of this complex to the nuclei. Also, ER stress correlates with altered plasticity of NG-108 cells. Indeed, involvement of the ER stress in morphological changes of differentiated NG-108 cells was verified by ER stressor thapsigargin, which generated similar morphological changes as haloperidol (Kubickova et al. 2016). Finally, nuclear calcium was increased, which might be due to the translocation of IP_3_R1s to the nucleus. Mitsuda and co-workers (2011) have shown that σ1Rs are transcriptionally upregulated via the PERK/eIF2a/ATF4 pathway and ameliorate cell death signaling. Miki and co-workers (2014) had reported that ER stress caused translocation of σ1Rs from cytoplasm to the nucleus. Function of σ1Rs in the nucleus should be further elucidated. Crottes and co-workers (2013) proposed functional consequences of such translocation. Because of the spatial dynamics of σ1Rs within the cell, the protein could also behave as a transcription factor that directly or indirectly controls a set of genes that encode ion channels. Many reports have shown the involvement of σ1R in a number of signaling pathways that potentially target transcriptional activity (e.g., MAP kinases, PKA, PI3 K/AKT, NFκ-B, c-Fos, CREB) (Crottes et al. 2013).

Another interesting issue is the mechanism of dopamine signaling. Haloperidol is primarily an antagonist of D2 receptors. These receptors generally transmit signal through Gi and inhibition of adenylyl cyclase. However, D1/D2 receptors can transmit signal through Gq and production of IP_3_ (for review see Beaulieu et al. 2015). We observed that D1/D2 heterodimers really occurred in a control group of differentiated NG-108 cells, but not in a group treated with haloperidol. Based on these results, we propose that due to block of D2 receptors by haloperidol, disintegration of D1/D2 complex occurs, and activity of the IP_3_R1 is significantly decreased due to a lack of IP_3_. Therefore, cells start to increase the IP_3_R1 expression. On the other hand, treatment with the σ1R antagonist BD 1047 did not lead to disintegration of D1/D2 complex. This observation would support the protective role of σ1Rs on IP_3_R1s, rather than its regulatory role.

An unexpected result was observed in our experiments. Nuclear calcium was significantly increased in BDµ/Xest-treated cells compared to cells treated with BDµ only. Additional experiments are needed to clarify this phenomenon. We propose that this change might be a compensatory mechanism involved in the regulation of transcription by σ1Rs.

We measured the length of neurites in differentiated cells following a 24-h treatment with either haloperidol or BD 1047, or after silencing σ1R. Haloperidol treatment for 24 h modulates the plasticity of differentiated NG-108 cells, and haloperidol and BD 1047 significantly increases the length of neurites and decreases their numbers per cell. Our observation does not agree with that of Ishima and Hashimoto (2012), who have shown that the potentiation of NGF-induced neurite outgrowth mediated by ifenprodil (a prototypical antagonist of the N-methyl-D-aspartate receptor) was significantly antagonized by the co-administration of the selective σ1R antagonist NE-100. The σ1R activation has been shown to promote neurite outgrowth in cerebellar granule neurons through the phosphorylation of tropomyosin receptor kinase B at Y515 (Kimura et al. 2013). From these results and from the literature (Kimura et al. 2013; Ishima et al. 2014), it is clear that σ1R affects cell plasticity. This demonstration of plasticity is dependent on the compound affecting the σ1Rs, the time and length of exposure and the differentiation status of the cells. Rather controversial results of various studies might originate from different cell types, affinity of ligands to σ1R, different concentrations of ligands used and methodology of neurite outgrowth assessment. Nevertheless, further studies on this issue are required.

In conclusion, haloperidol treatment causes disruption of D1/D2 heterodimer and suppression of the IP_3_R activity. This probably leads to an increase of IP_3_R1 expression, depletion of calcium from ER, which generates ER stress. As a consequence, σ1Rs are also upregulated. Both IP3R1 and σ1Rs form a cluster and translocate to the nucleus, where they increase the level of intranuclear calcium. In differentiated NG-108 cells, this process is likely to result in changes to neuronal plasticity.

## Abbreviations

BD: *N*-[2-(3,4-Dichlorophenyl)ethyl]-*N*-methyl-2-(dimethylamino)ethylamine dihydrobromide
dbcAMP: N6,2′-O-Dibutyryladenosine 3′,5′-cyclic monophosphate sodium salt
DMEM: Minimal Essential Medium of Dulbecco
ER: Endoplasmatic reticulum
Fluo-3AM: (4-(6– Acetoxymethoxy-2,7–dichloro–3–oxo–9- xanthenyl)-4′-methyl -2,2′(ethylenedioxy) dianiline- N,N,N′,N′-tetraacetic acid tetrakis (acetoxymethyl) ester
H: Haloperidol
IP_3_R (1–3): Inositol 1,4,5-trisphosphate receptor (type 1–3)
PRE-084: 2-(4-Morpholinethyl) 1- phenylcyclohexane-carboxylate hydrochloride
SA 4503: 1-[2-(3,4-Dimethoxyphenyl)ethyl]-4-(3-phenylpropyl)- piperazine dihydrochloride
Xest: Xestospongin C
σ1Rs: Sigma 1 receptors
